# Supplementary material for: Hypertension Self-Management and Stroke Recovery Among Rural Adults in the Stroke Belt: A Mixed-Methods Study
Source: medRxiv. 2025 Jun 13:2025.06.12.25329283. Preprint. [Version 1] doi: 10.1101/2025.06.12.25329283 (PMC12204430; doi:10.1101/2025.06.12.25329283)
Supplement: Supplement 1 [file NIHPP2025.06.12.25329283v1-supplement-1.pdf]

medRxiv preprint doi: https://doi.org/10.1101/2025.06.12.25329283; this version posted June 13, 2025. The copyright holder for this preprint (which was not certified by peer review) is the author/funder, who has granted medRxiv a license to display the preprint in perpetuity.
This article is a US Government work. It is not subject to copyright under 17 USC 105 and is also made available for use under a CC0 license.

Supplemental Table 1: Semi –structured interview guide

Sample interview questions	Example of probes
Can you tell me a bit about the programs or services you have found the most helpful for you during your recovery journey	How about during your/their transition from the hospital or rehab to home? After your/their transition to home? During later stages of your/their recovery?
Are there specific people you connect with or places you go to look online or otherwise for services.?	How about during the transition to home? After the transition to home? In the hospital? What services/services have helped you to find information you needed? Can you tell me a bit about what you think would make finding the information you need easier? Phone, group, internet, website, service, other?
Did you receive any informational activities/ materials related to your stroke condition or management of life after stroke and management of its risk factors?	Was what you worked on useful for you? Was there educational information relevant to you? What was missing? What other components could have been in that program? Like exercise, education related to risks like high BP, medication adherence, stress management, fall and fatigue prevention and management, healthy diet management, management of activity of daily living etc.?
Can you talk about finding ways to perform the activities: Personal strategies to deal with the day.	In the past month, because of your condition, were there things that you did to stay healthy? For example, did you pay specific attention to your diet and/or exercise? In the past month, because of your condition, have you used any deliberate strategies to manage your day to day activities at home, at work or in the community? For example, you took frequent rests? Planned your days? Asked for help? Shortened your work hours? Worked from home? Tell me about those strategies. Prioritized your time with your family or friends? Choose your friends carefully? Tell me about those strategies.
Can you tell me a bit about any other areas in your life where you need extra support?	How the services you received or programs you participated in suited your unique needs?

medRxiv preprint doi: https://doi.org/10.1101/2025.06.12.25329283; this version posted June 13, 2025. The copyright holder for this preprint (which was not certified by peer review) is the author/funder, who has granted medRxiv a license to display the preprint in perpetuity.
This article is a US Government work. It is not subject to copyright under 17 USC 105 and is also made available for use under a CC0 license.

	Appropriateness and tailoring to situation/culture/language/age?
